# Development and evaluation of a search filter to identify prognostic factor studies in Ovid MEDLINE

**DOI:** 10.1186/s12874-022-01595-9

**Published:** 2022-04-10

**Authors:** Elena Stallings, Andrea Gaetano-Gil, Noelia Alvarez-Diaz, Ivan Solà, Jesús López-Alcalde, Daniel Molano, Javier Zamora

**Affiliations:** 1 Clinical Biostatistics Unit, Instituto Ramón Y Cajal de Investigación Sanitaria, 28034 Madrid, Spain; 2 CIBER Epidemiología Y Salud Pública (CIBERESP), 28029 Madrid, Spain; 3 Library, Hospital Universitario Ramón Y Cajal (IRYCIS), Madrid, Spain; 4 Sant Pau Biomedical Research Institute (IIB Sant Pau), 08041 Barcelona, Spain; 5 Faculty of Health Sciences, Universidad Francisco de Vitoria, 28223 Madrid, Spain; 6 Institute for Complementary and Integrative Medicine, University Hospital Zurich and University of Zurich, CH-8091 Zurich, Switzerland; 7 Department of Critical Care, Fundacion Universitaria de Ciencias de La Salud, Hospital de San José, Carrera 19 # 8-32, 11001 Bogota, Colombia; 8 Institute of Metabolism and Systems Research, WHO Collaborating Centre for Global Women’s Health, University of Birmingham, Birmingham, B15 2TT UK

**Keywords:** Prognostic factor, Search filter, Systematic review

## Abstract

**Background:**

Systematic reviews (SRs) are valuable resources as they address specific clinical questions by summarizing all existing relevant studies. However, finding all information to include in systematic reviews can be challenging. Methodological search filters have been developed to find articles related to specific clinical questions. To our knowledge, no filter exists for finding studies on the role of prognostic factor (PF). We aimed to develop and evaluate a search filter to identify PF studies in Ovid MEDLINE that has maximum sensitivity.

**Methods:**

We followed current recommendations for the development of a search filter by first identifying a reference set of PF studies included in relevant systematic reviews on the topic, and by selecting search terms using a word frequency analysis complemented with an expert panel discussion. We evaluated filter performance using the relative recall methodology.

**Results:**

We constructed a reference set of 73 studies included in six systematic reviews from a larger sample. After completing a word frequency analysis using the reference set studies, we compiled a list of 80 of the frequent methodological terms. This list of terms was evaluated by the Delphi panel for inclusion in the filter, resulting in a final set of 8 appropriate terms. The consecutive connection of these terms with the Boolean operator OR produced the filter. We then evaluated the filter using the relative recall method against the reference set, comparing the references included in the SRs with our new search using the filter. The overall sensitivity of the filter was calculated to be 95%, while the overall specificity was 41%. The precision of the filter varied considerably, ranging from 0.36 to 17%. The NNR (number needed to read) value varied largely from 6 to 278. The time saved by using the filter ranged from 13–70%.

**Conclusions:**

We developed a search filter for OVID-Medline with acceptable performance that could be used in systematic reviews of PF studies. Using this filter could save as much as 40% of the title and abstract screening task. The specificity of the filter could be improved by defining additional terms to be included, although it is important to evaluate any modification to guarantee the filter is still highly sensitive.

**Supplementary Information:**

The online version contains supplementary material available at 10.1186/s12874-022-01595-9.

## Introduction

It is essential to carry out a systematic and extensive search for any type of systematic review (SR). However, searches can often retrieve an overwhelming number of studies [1, 2]. To overcome this, methodological search filters have been developed to find articles related to specific clinical questions. A search filter is a pre-defined combination of search terms combined into a search strategy using the “AND” Boolean operator. Dozens of search filters exist for retrieving randomized controlled trials (RCTs) [3, 4]. These filters have been successful in reducing the number of references needed to screen in SRs, however this is difficult to reproduce for prognostic factor studies, as the literature pertaining to non-interventional studies is more variable. Unlike RCTs, non-interventional investigations have heterogenous, non-standardized study designs [5]. These studies also suffer from poorer indexing of terms, thus making them more difficult to find in the database. Due to these limitations, the use of filters in diagnostic or prognostic studies is not widely recommended [6–8].

Prognosis research focuses on identifying variables that allow the estimation of the possibilities of improvement or worsening of a given health problem. This area of clinical research is becoming significantly more important, as throughout the world, people are living longer, but with more chronic health conditions and diseases. Prognosis research can be classified into four different themes or areas of research: fundamental prognostics, prognostic models, stratified medicine, and prognostic factors [9–12]. A prognostic factor (PF) “is any measure that, among people with a given health condition (that is, a start point), is associated with a subsequent clinical outcome (an endpoint)” [11]. Generic filters exist for finding prediction and prognosis studies such as the Haynes broad filter, Ingui filter and the Yale prognosis and natural history filter [13–15]. These published prognostic search filters have lower sensitivity and precision than other types of search filters such as those for medical intervention studies [16]. While carrying out various PF systematic reviews we explored the possibility of using a PF filter [17, 18], however, to the best of our knowledge, no filter exists for these studies. The aim of this paper is to develop and evaluate a search filter for prognostic factor studies to be used in SRs. The main objective of the filter is to achieve maximum sensitivity so as not to lose any relevant studies when using the filter, while maintaining specificity to make the search more efficient.

## Methods

We developed a search filter partially based on methods described by Rietjens et al., Sampson et al., and also on the criteria of the filter appraisal tool developed by Glanville et al. [19–21]. The completed filter appraisal checklist is available as supplementary material. We completed the study in three phases as outlined below:

1. Identification of a reference set (relative recall)
2. Search term selection
3. Filter evaluation

### Identification of a reference set (relative recall)

The first step of search filter development is to create the reference set list, which is most often referred to as the gold standard [22]. The reference set is a known set of studies that are relevant to the general type of studies under review, in our case, prognostic factor studies. We used the relative recall method, which involves replicating the searches of systematic reviews and using the included studies in these reviews as the reference standard [21]. Relative recall is useful as it allows for the inclusion of a broader range of journals and publication years than otherwise could be included practically by manual searching [7, 21]. This approach is also more generalizable to topics that are important for our filter, as the literature is spread across a broad range of journals.

We searched for prognostic factor systematic reviews in PubMed by combining the filter for systematic reviews from “National Library of Medicine: systematic reviews PubMed subset strategy [2018] [PubMed]” and “Prognostic factors [title]. Then these reviews were screened to see if they met the criteria for inclusion in the gold standard. These criteria were that they carried out a search on Ovid MEDLINE, did not include a prognosis filter or prognosis terms in the search strategy, and that they used a search strategy that was publicly available and reproducible. Additionally, we made sure that the SR´s were related to different clinical topics to allow for generalizability.

### Search term selection

#### Frequency analysis

Search term selection was partially based on the objective method used by Rietjens 2019 [20]. A word frequency analysis of titles and abstracts of PF articles was carried out using the free online software systematic review accelerator [23]. We separately analyzed the language of both the included and excluded studies of the SRs used for relative recall to create two distinct lists of terms.

#### Calculate chi square values

Chi square values were calculated for terms generated from the word frequency analysis. From this, we determined the significance of the difference in relative frequencies of the terms in positive studies (the studies that are included in the review) and negative studies (studies not included in the review). As expected, given the small number of studies included, all terms showed non-significant results. Thus, we complemented this frequency analysis with a Delphi panel of experts to reach a consensus on the terms selected for the filter.

#### Delphi panel

The Delphi panel consisted of 15 members of various specialties, in particular systematic reviewers, statisticians, clinicians and information retrieval specialists. Each panelist had to evaluate the appropriateness of including each term in the filter. We used the RAND definitions of agreement to classify the terms as appropriate, neutral or inappropriate for use in the filter and also to decide whether this qualification was agreed on by a majority of the panel members [24]. The Delphi method consisted of three rounds, the first two being individual ratings and the last round was a panel meeting where a discussion took place on the ratings given to each term. The most relevant methodological terms were extracted from the frequency analysis and made into a list of 80 terms. This list was given to the panel to rate on a scale of 1–9, with 1 being the least appropriate for inclusion and 9 being most appropriate. The terms scoring between 7 and 9 on the Delphi were defined as potentially eligible for inclusion in the filter [24]. The consecutive connection of these terms with the Boolean operator OR produced the final search strategy (filter).

### Filter evaluation

An essential component of the search filter development process is the evaluation of how well the search filter performs in retrieving relevant records in a systematic review. To carry out the evaluation the filter was combined using the Boolean operator AND with the broad search strategy for Ovid MEDLINE that was used in each included SR.

During the evaluation we tested the sensitivity, specificity, precision, and number needed to read (NNR) of the filter. We used Table 1 below to guide us in the evaluation:

**Table 1:** Table to calculate sensitivity, specificity, precision and NNR of the filter

	Reference set articles	Non-reference set articles
Retrieved	**A** (True Positive)	**B** (False Positive)
Not retrieved	**C** (False Negative)	**D** (True Negative)

Sensitivity is the proportion of the total number of references included in the reference set retrieved by the filter [25–27]. If the search had low sensitivity, it would miss a large proportion of relevant articles. In contrast, a highly sensitive search is constructed so that it can pick up most of the relevant articles. It was calculated by: **(A/(A + C)) × 100.**

Specificity is the proportion of the total number of non- relevant references that are not retrieved by the filter [25, 27]. It was calculated by: **(D/(B + D)) × 100.**

Precision (or positive predictive value PPV) is the number of relevant records retrieved as a proportion of the total number of records retrieved by the filter [25, 27]. It was calculated by: **(A/(A + B)) × 100.**

The number needed-to read (NNR) is a measure of the usability of the filter, as it indicates how many records a searcher must screen for each relevant record retrieved [25–27]. In the context of searching, NNR refers to the number of references that have to be screened to find one additional relevant article. A relatively high NNR means a lot of references would have to be screened, thus having important resource implications in terms of time and cost, whereas a low NNR means that relevant articles can be identified quicker without having to screen large numbers of titles and abstracts. It was calculated by: **(1/precision) × 100.**

The measure of time saved is the percentage of studies that could be screened but can be saved by using the filter. When using the filter, as compared to without the filter, less articles should be retrieved thus saving time during the screening process. It was calculated by: **((C + D)/(A + B + C + D)) × 100.**

Table 2 provides a summary of the different performance measures and formulas used in our study.

**Table 2:** Summary of performance measures and formulas

Performance measure	Formula
Sensitivity	(A/(A + C)) × 100
Specificity	(D/(B + D)) × 100
Precision	(A/(A + B)) × 100
Number needed to read (NNR)	(1/precision) × 100
Time saved	((C + D)/(A + B + C + D)) × 100

We computed a pooled average of sensitivity, and specificity over the 6 reviews used for evaluation by means of a random effects meta-analysis of proportions using Stata v. 16.0 [28].

## Results

### Identification of a reference set (relative recall)

**Fig. 1:**
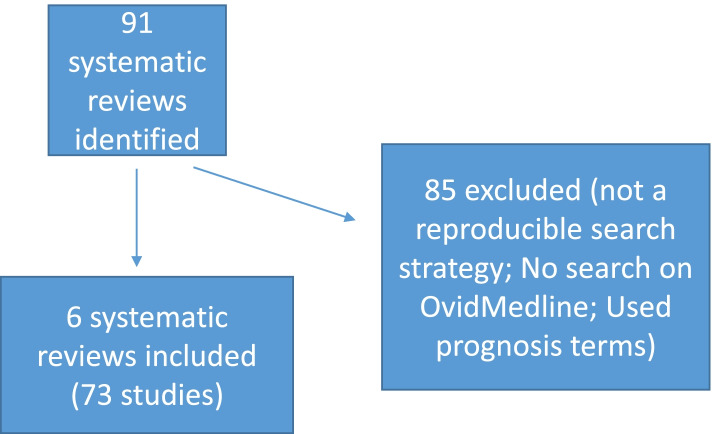
Flow diagram of reference set search

As outlined in Fig. 1, our search on PubMed yielded ninety-one SRs of prognostic factors of various topics. We excluded eighty-five SRs due to not having a publicly available and reproducible search strategy, not having carried out a search on Ovid MEDLINE, or for having used prognosis terms in the search strategy. Finally, we formed our reference set with six SRs that met all of our criteria [29–34]. Each individual reference set included between 3 and 22 studies. The studies from the 6 individual reference sets were combined into one overall reference set with a total of 73 studies.. The prognostic factors assessed in these reviews were the following: symptoms of depression, protease activity, sarcopenia, interstitial pneumonia, controlling nutritional status score, and interim PET results.

### Selection of search terms

After completing the word frequency analysis, we compiled a list of 80 of the most frequent methodological terms in the prognostic factor reference set. This list of terms was evaluated by the Delphi panel for inclusion in the filter. At the end of the last round of the Delphi we had a list of 8 terms which were deemed appropriate and agreed upon by the panel to include in the filter. We consulted the information retrieval specialists from the Delphi panel about the best way to combine them using MeSH and free text title/abstract terms. We truncated the terms prognostic (prognos*) and predictive (predict*) to be as inclusive as possible in the search. The consecutive connection of these terms with the Boolean operator OR produced the final search strategy (filter) and it is shown below in Table 3.

**Table 3:** Terms included in prognostic factor filter

1 exp Risk/
2 risk.tw
3 exp Cohort Studies/
4 cohort.tw
5 exp Prognosis/
6 "prognos*".tw
7 "predict*".tw
8 exp Incidence/
9 incidence.tw
10 exp Survival Analysis/
11 survival.tw
12 "causal factor".tw
13 course.tw
14 or/1–13

### Filter evaluation

We evaluated the filter using the relative recall method with the six systematic reviews in our reference set. The filter was added to the end of the search strategy of each SR using the Boolean operator “AND”. The complete search strategy was entered into Ovid MEDLINE and the number of references retrieved was recorded and downloaded into Endnote [35]. To measure the performance of the filter we compared the references retrieved from the original search in the review with our new search using the filter. The performance of the filter in each review is shown in Table 4.

**Table 4:** Results for sensitivity, specificity, precision, NNR and NNS of the filter evaluated in each review

Study	Number of included studies in SR	Number of studies retrieved in original SR search	Sensitivity (%)	Specificity (%)	Precision (%)	NNR	Time saved (%)
Kamiya [33]	12	120	100	46	17	6	42
Pinheiro [34]	3	1314	100	37	0.4	278	37
Westby [29]	16	784	31.25	70	2	47	70
Aldin [30]	22	5562	100	35	0.6	164	35
Yang [32]	13	565	100	44	4	26	41
Takagi [31]	7	100	100	14	8	12	13

*SR* systematic review, *NNR* number needed to read

**Fig. 2:**
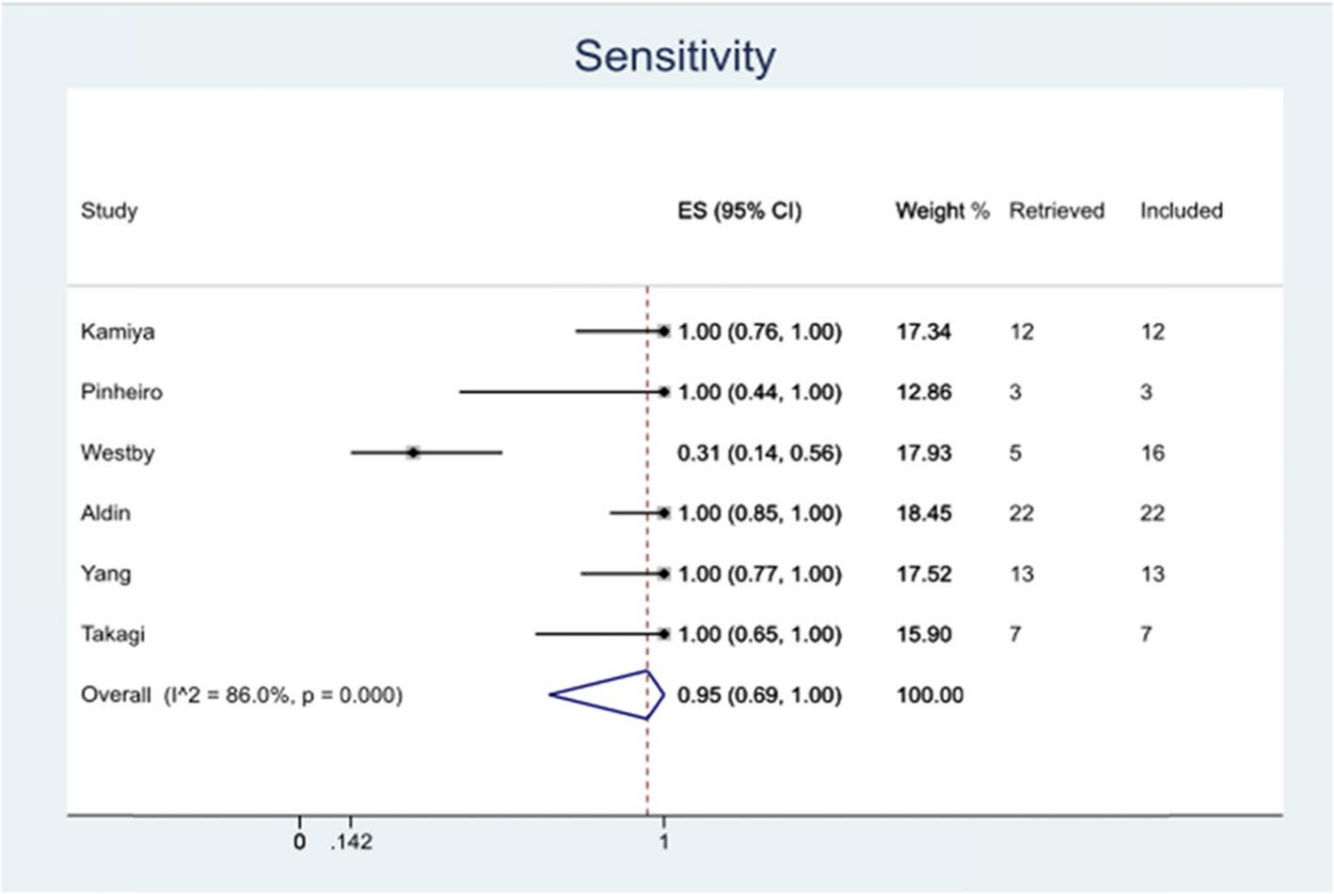
Sensitivity of the filter in various systematic review searches

The filter had a sensitivity of 100% in all reference sets except for Westby 2018 [29], which had a low sensitivity of 31%. As can be seen below in Fig. 2, the filters overall sensitivity was calculated to be 95% (95% CI 69%-100%).

**Fig. 3:**
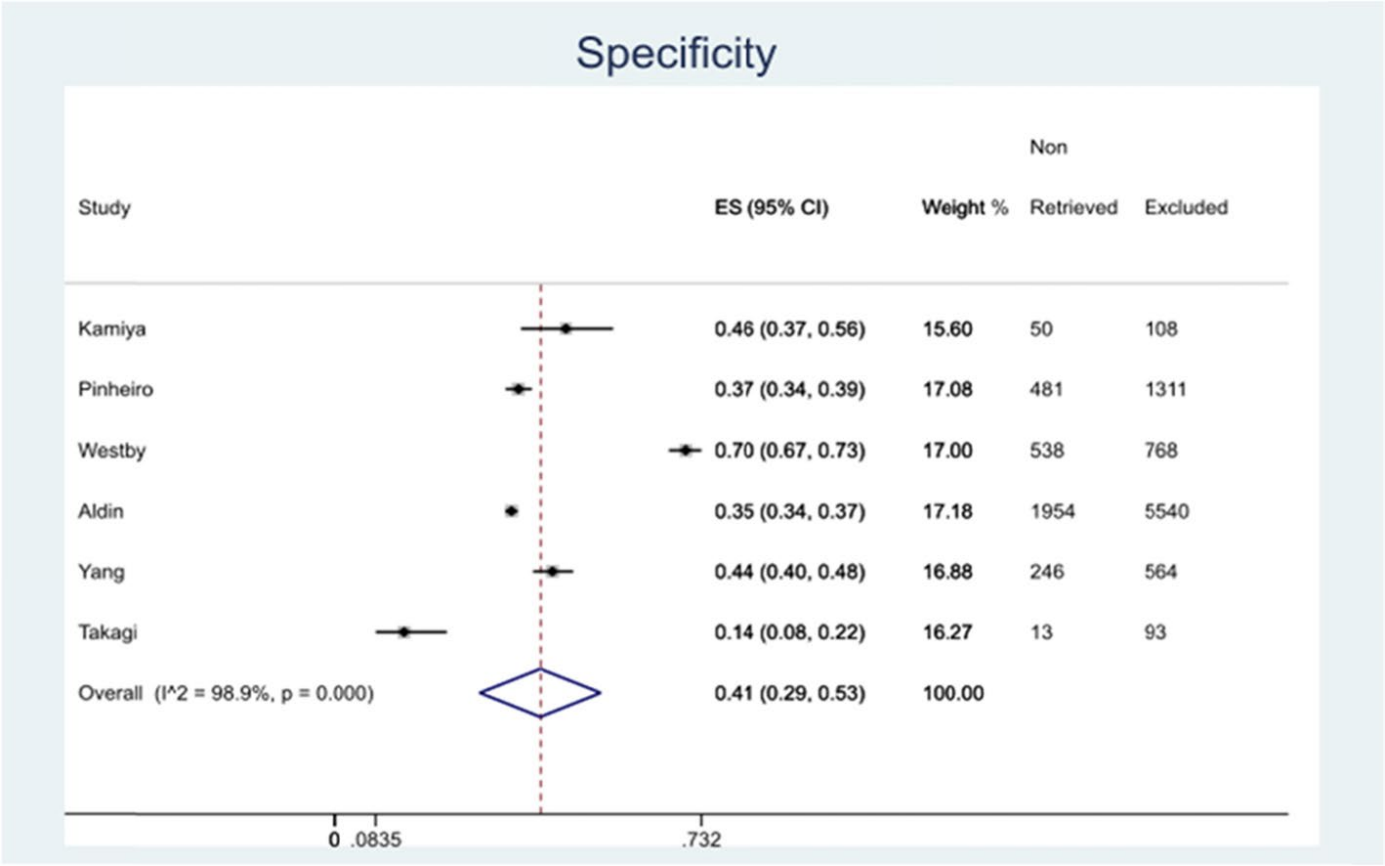
Specificity of filter in various systematic review searches

The specificity varied from 14–70%, with the highest performance of specificity being in Westby 2018 [29] and the lowest in Takagi 2019 [31]. As seen below in Fig. 3, the overall specificity was calculated to be 41% (95% CI 29–43%). The precision performance also varied considerably ranging from 0.4 [34] to 17% [33]. The NNR value varied largely among reviews ranging from 6 to 278. Time saving was substantial ranging from 13% (Takagi [31]) to 70% (Westby [29]).

## Discussion

### Main findings

We aimed to develop and test a search filter for finding studies about the role of PFs in Ovid MEDLINE. Overall, the obtained filter showed an excellent sensitivity to retrieve studies from a reference set constructed from studies included in relevant systematic reviews in the field. Specificity was much lower with an overall combined specificity of 41%. Precision ranged from 0.36 to 17%, but it is important to note that efforts to optimise recall has a direct impact on the screening burden (total number of references retrieved) and may not be an appropriate indicator to measure performance of approaches focusing on sensitivity. Resulting from these statistics, the number of references required to screen to retrieve a relevant article varied hugely, from 6 to 277. We calculated that, when using the filter, the time for screening would be lower in all reference sets (13 to 70%).

Out of the six reviews in which we tested the filter, Westby 2018 [29] was the only review where the filter was not effective in retrieving all of the reference set studies. It was a Cochrane review on protease activity as a prognostic factor for healing wounds [29]. After examining the studies that weren´t retrieved, we observed that they did not use any of the search terms attributable to prognosis and their approach was not obvious for usual prognostic factor studies. Those studies had terms such as influence or associated that could make them in some way related to prognosis. Another possible explanation for the low sensitivity in Westby 2018 [29] could be that the review authors were generous or lenient with the studies that they included in the review as they had broad inclusion criteria for the studies such as including prognostic factor studies and prediction model development studies and including studies with any period of follow-up. When examining the flow diagram of Westby 2018 [29], they screened a lot of full texts (10% of the titles and abstracts screened were passed on to the full text stage). In comparison, most of the other reviews in the reference set only passed on 2–3% of studies to the full text stage, thus they were seemingly stricter with the prognostic factor study criteria. When we added our filter to the other systematic review strategies, the sensitivity was 100%, as all included studies were retrieved.

### Comparison with available prognosis filters

There are a few published filters for prognosis studies which focus on prognostic models and prediction rules. We compared our prognostic factor filter with the Haynes broad prognosis filter [14]: (incidence[MeSH:noexp] OR mortality[MeSH Terms] OR follow up studies[MeSH:noexp] OR prognos*[Text Word] OR predict*[Text Word] OR course*[Text Word]). We chose this filter as a comparison since it is the one that is most available to people who use PubMed. In general, the filter is known to have a sensitivity of 90% and specificity of 80%. We evaluated this filter in our reference set. As can be seen below in Table 5 the filter was less sensitive overall than our PF filter (74% 95%CI (0.45—0.96)), but it was more specific (0.63 95%CI (0.51—0.74)). All of the SR´s in our reference set had a similar precision performance as the Haynes filter. This is because the reference sets had very low numbers of included studies, which this statistic is dependent on. More time can be saved using the Haynes filter, but that is at a risk of losing potentially relevant studies to include in the review.

**Table 5:** Results from Haynes sensitive broad filter in our reference set

Study	Sensitivity (%)	Specificity (%)	Precision (%)	NNR	Time saved (%)
Kamiya [33]	75	63	18.4	5	59
Pinheiro [34]	100	67	0.7	146	67
Westby [29]	13	87	1.9	52	87
Aldin [30]	80	56	1.5	68	56
Yang [36]	92	62	5.4	19	60
Takagi [31]	86	35	9.1	11	34

*NNR* number needed to read

### Strengths and limitations

Our relative recall references included various topics, thus allowing us to evaluate the filter over many different clinical situations. If the references in the reference set are from one area only it can lead to subject bias in the filter (working well in some subjects, but not others). Through using the relative recall method, we were able to ensure that each study in the reference set was in fact a prognostic factor study. It can be difficult to decipher prognostic factor studies from other studies at times, so since we were using studies that were included in prognostic factor systematic reviews, we could be assured that they were truly prognostic factor studies.

An important limitation to note is that the reference standard contained a low number of systematic reviews, which in turn contained a low number of studies (73). This is because prognostic factor studies and thus prognostic factor systematic reviews are a relatively new area of research. For example, in the Cochrane library there are 10 prognosis systematic reviews, while there are 8,487 intervention systematic reviews.

When developing the protocol for this study, we realized that there were many different methods that researchers have used in the past to create a search filter. We examined all the published methods and weighed up our options before deciding on which methods to follow. If we had more resources, time, and manpower available there are more robust methods that we could employ in the future. These other methods include creating a larger reference set of PF studies or creating a traditional gold standard through manually searching for studies. However, even though we had a small reference set of PF studies the filter can still be considered a 3rd generation search filter. Jenkins et al. describe 3rd generation filters as the most objective filter as “terms may be derived objectively through a frequency analysis of relevant records and combined on either the basis of their individual or overall performance or through statistical analysis” [22].

### Implications for research

This filter has a high sensitivity so we can be assured that the risk of missing a study is very low. However, as we noted with the studies in Westby 2018 [29], not all PF studies include typical prognostic words, so we still need to think carefully about what kind of studies we might be searching for and if they will include the correct terms. The use of the filter in search strategies could decrease the number of studies needed to be manually screened. Many times, search strategies for PF systematic reviews yield large numbers of studies from the search, for example 20,000–100,000 references. Thus, it can take a lot of time) and resources to screen through them all, making the NNR an important statistic. This PF filter needs to be evaluated in rapid reviews, as time constraints in these reviews make efficient searches even more necessary.

### Future research

Evaluating the performance of the search filter against a reference set that is different from the one used to identify the search terms can lead to a search filter of higher quality. As PF research increases, we expect to see many more studies being available for use in the validation process. In the future, to improve the quality of the filter, we would like to validate it using a new reference set of PF studies.

### Conclusions

To the best of our knowledge, no search filter exists for locating PF studies in Ovid MEDLINE, nor in any other online database. Our filter had a high sensitivity of 95% overall in the systematic reviews in which we tested it. Its specificity on the other hand, was lower at 41% overall. Our aim was to create a sensitive filter as we feel the most important part of search filter development is to not lose any relevant studies in the search. Further research is still needed on this topic to increase the specificity of the filter, while keeping its high sensitivity.

## Supplementary Information

**Additional file 1.**  Appraisal checklist. 

**Additional file 2.** Terms analysed in word frequency analysis.

**Additional file 3.** Delphi Panel Results.

## Data Availability

The datasets used and/or analysed during the current study are available from the corresponding author on reasonable request.

## Abbreviations

SR: Systematic review
PF: Prognostic factor
RCT: Randomized controlled trial
NNR: Number needed to read

## References

[1] Prady SL, Uphoff EP, Power M, Golder S (2018). Development and validation of a search filter to identify equity-focused studies: reducing the number needed to screen. BMC Med Res Methodol.

[2] Shariff SZ, Sontrop JM, Haynes RB, Iansavichus AV, McKibbon KA, Wilczynski NL (2012). Impact of PubMed search filters on the retrieval of evidence by physicians. CMAJ.

[3] Glanville J, Dooley G, Wisniewski S, Foxlee R, Noel-Storr A (2019). Development of a search filter to identify reports of controlled clinical trials within CINAHL Plus. Health Info Libr J.

[4] Glanville J, Kotas E, Featherstone R, Dooley G (2020). Which are the most sensitive search filters to identify randomized controlled trials in MEDLINE?. J Med Libr Assoc.

[5] Tugwell P, Knottnerus JA (2017). Current study design labels are confusing! Call for consensus on better terminology that clearly reflects specific features. J Clin Epidemiol.

[6] Kastner M, Wilczynski NL, McKibbon AK, Garg AX, Haynes RB. Diagnostic test systematic reviews: bibliographic search filters (“Clinical Queries”) for diagnostic accuracy studies perform well. J Clin Epidemiol. 2009;62(9):974–81. 10.1016/j.jclinepi.2008.11.006 PMC2737707 19230607

[7] Beynon R, Leeflang MM, McDonald S, Eisinga A, Mitchell RL, Whiting P (2013). Search strategies to identify diagnostic accuracy studies in MEDLINE and EMBASE. Cochrane Database Syst Rev..

[8] Boulos L, Ogilvie R, Hayden JA (2021). Search methods for prognostic factor systematic reviews: a methodologic investigation. J Med Libr Assoc.

[9] Hemingway H, Croft P, Perel P, Hayden JA, Abrams K, Timmis A (2013). Prognosis research strategy (PROGRESS) 1: a framework for researching clinical outcomes. BMJ.

[10] Hingorani AD, Windt DA, Riley RD, Abrams K, Moons KG, Steyerberg EW (2013). Prognosis research strategy (PROGRESS) 4: stratified medicine research. BMJ.

[11] Riley RD, Hayden JA, Steyerberg EW, Moons KG, Abrams K, Kyzas PA (2013). Prognosis Research Strategy (PROGRESS) 2: prognostic factor research. PLoS Med.

[12] Steyerberg EW, Moons KG, van der Windt DA, Hayden JA, Perel P, Schroter S (2013). Prognosis Research Strategy (PROGRESS) 3: prognostic model research. PLoS Med.

[13] Geersing GJ, Bouwmeester W, Zuithoff P, Spijker R, Leeflang M, Moons KG (2012). Search filters for finding prognostic and diagnostic prediction studies in Medline to enhance systematic reviews. PLoS One.

[14] Wilczynski NL, Haynes RB (2004). Developing optimal search strategies for detecting clinically sound prognostic studies in MEDLINE: an analytic survey. BMC Med.

[15] Ingui BJ, Rogers MA (2001). Searching for clinical prediction rules in MEDLINE. J Am Med Inform Assoc.

[16] Chatterley T, Dennett L (2012). Utilisation of search filters in systematic reviews of prognosis questions. Health Info Libr J.

[17] López-Alcalde J, Stallings EC, Zamora J, Muriel A, van Doorn S, Alvarez-Diaz N, Fernandez-Felix BM, Quezada Loaiza CA, Perez R, Jimenez D. Sex as a prognostic factor for mortality in adults with acute symptomatic pulmonary embolism (Protocol). Cochrane Database Syst Rev. 2021;(Issue 1). Art. No.: CD013835. 10.1002/14651858.CD013835. 10.1002/14651858.CD013835.pub2 PMC12043200 40110896

[18] Lopez-Alcalde J, Antequera Martín A, Stallings E, Muriel A, Fernández-Félix B, Solà I (2020). Evaluation of the role of sex as a prognostic factor in critically ill adults with sepsis: systematic review protocol. BMJ Open.

[19] Glanville J, Bayliss S, Booth A, Dundar Y, Fernandes H, Fleeman ND (2008). So many filters, so little time: the development of a search filter appraisal checklist. J Med Libr Assoc.

[20] Rietjens JA, Bramer WM, Geijteman EC, van der Heide A, Oldenmenger WH (2019). Development and validation of search filters to find articles on palliative care in bibliographic databases. Palliat Med.

[21] Sampson M, Zhang L, Morrison A, Barrowman NJ, Clifford TJ, Platt RW (2006). An alternative to the hand searching gold standard: validating methodological search filters using relative recall. BMC Med Res Methodol.

[22] Jenkins M (2004). Evaluation of methodological search filters–a review. Health Info Libr J.

[23] University B. Systematic review accelerator: Institute for Evidence Based Healthcare; 2021. Available from: https://sr-accelerator.com/#/.

[24] Fitch K, Bernstein SJ, Aguilar MD, Burnand B, LaCalle JR. The RAND/UCLA appropriateness method user's manual. California: Rand Corp Santa Monica CA; 2001.

[25] van de Glind EM, van Munster BC, Spijker R, Scholten RJ, Hooft L (2012). Search filters to identify geriatric medicine in Medline. J Am Med Inform Assoc.

[26] Kok R, Verbeek JA, Faber B, van Dijk FJ, Hoving JL (2015). A search strategy to identify studies on the prognosis of work disability: a diagnostic test framework. BMJ Open.

[27] Cooper C, Varley-Campbell J, Booth A, Britten N, Garside R (2018). Systematic review identifies six metrics and one method for assessing literature search effectiveness but no consensus on appropriate use. J Clin Epidemiol.

[28] StataCorp (2019). Stata Statistical Software: Release 16.

[29] Westby MJ, Dumville JC, Stubbs N, Norman G, Wong JK, Cullum N (2018). Protease activity as a prognostic factor for wound healing in venous leg ulcers. Cochrane Database Syst Rev.

[30] Aldin A, Umlauff L, Estcourt LJ, Collins G, Moons KG, Engert A (2019). Interim PET-results for prognosis in adults with Hodgkin lymphoma: a systematic review and meta-analysis of prognostic factor studies. Cochrane Database Syst Rev..

[31] Takagi K, Domagala P, Polak WG, Buettner S, Ijzermans JNM (2019). Prognostic significance of the controlling nutritional status (CONUT) score in patients undergoing hepatectomy for hepatocellular carcinoma: a systematic review and meta-analysis. BMC Gastroenterol.

[32] Yang M, Shen Y, Tan L, Li W (2019). Prognostic Value of Sarcopenia in Lung Cancer: A Systematic Review and Meta-analysis. Chest.

[33] Kamiya H, Panlaqui OM (2019). Systematic review and meta-analysis of the prognosis and prognostic factors of interstitial pneumonia with autoimmune features. BMJ Open..

[34] Pinheiro MB, Ferreira ML, Refshauge K, Maher CG, Ordoñana JR, Andrade TB (2016). Symptoms of depression as a prognostic factor for low back pain: a systematic review. Spine J.

[35] Clarivate A (2016). EndNote version X7.7.1, software for reference management.

[36] Bhojwani D, Yang JJ, Pui CH (2015). Biology of childhood acute lymphoblastic leukemia. Pediatr Clin North Am.

