# Supplementary material for: Development and evaluation of a search filter to identify prognostic factor studies in Ovid MEDLINE
Source: BMC Med Res Methodol. 2022 Apr 10;22:107. doi: 10.1186/s12874-022-01595-9 (PMC8996648; doi:10.1186/s12874-022-01595-9)
Supplement: Supplementary file 1 — Additional file 1. Appraisal checklist. [file 12874_2022_1595_MOESM1_ESM.pdf]

A. Information	Answers
A.1. State the author's objective.	To create and validate a sensitive search filter for prognostic factor studies
A.2. State the focus of the research.	**[x] Sensitivity-maximizing**<br>[ ] Precision-maximizing<br>[ ] Specificity-maximizing<br>[ ] Balance of sensitivity and specificity/precision<br>**[x] Other… also to reduce the NNR**
A.3. Database(s) and search interface(s).	Ovid MEDLINE
A.4. Describe the methodological focus of the filter (e.g., RCTs).	Prognostic factor studies
A.5. Describe any other topic that forms an additional focus of the filter (e.g., clinical topics such as breast cancer, geographic location such as Asia, or population grouping such as paediatrics).	Not applicable
A.6. Other observations.	None
**B. Identification of a gold standard (GS) of known relevant records**	
B.1. Did the authors identify 1 or more gold standards (GSs)? None/1/2/3/4/5/More than 5	6
B.2. How did the authors identify the records in each GS?	Included studies of prognostic factor systematic reviews
B.3. Report the dates of the records in each GS.	
B.4. What are the inclusion criteria for each GS?	To be an included study in systematic review of prognostic factors with a reproducible search strategy
B.5. Describe the size of each GS and the authors' justification, if provided (e.g., the size of the GS may have been determined by a power calculation).	
B.6. Are there limitations to the gold standard(s)? Yes/No/Unclear	
B.7. How was each GS used?	[x] To identify potential search terms<br>[ ] To derive potential strategies (groups of terms)<br>[x] To test internal validity<br>[ ] To test external validity<br>[ ] Other, please specify
B.8. Other observations.	
**C. How did the researchers identify the search terms in their filter(s)? (Select all that apply)**	
C.1. Adapted a published search strategy. Yes/No/Unclear (please describe)	no
C.2. Asked experts for suggestions of relevant terms. Yes/No/Unclear (please describe)	Yes, with the use of a Delphi panel
C.3. Used a database thesaurus. Yes/No/Unclear (please describe)	no
C.4. Performed statistical analysis of terms in a GS set of records (see B above). Yes/No/Unclear (please describe)	no
C.5. Extracted terms from the GS set of records (see B above). Yes/No/Unclear (please describe)	yes
C.6. Extracted terms from some relevant records (but not a GS). Yes/No/Unclear (please describe)	Yes- chi squared values calculated
C.7. Tick all types of search terms tested.	[x] Subject headings<br>[x] Text words (e.g. in title, abstract)<br>[ ] Publication types<br>[ ] Subheadings<br>[ ] Check tags<br>[ ] Other, please specify
C.8. Include the citation of any adapted strategies.	Not applicable
C.9. How were the (final) combination(s) of search terms selected?	Through the Delphi panel
C.10. Were the search terms combined (using Boolean logic) in a way that is likely to retrieve the studies of interest?	Combined using OR to maximise sensitivity
C.11. Other observations.	
**D. Internal validity testing (This type of testing is possible when the search filter terms were developed from a known GS set of records.)**	
D.1. How many filters were tested for internal validity?	1
For each filter report the following information.	
D.2. Was the performance of the search filter tested on the GS from which it was derived? Yes/No/Unclear (please describe)	Yes, it was tested on the same reference standards of which the search terms where derived.

D.3. Report sensitivity data (a single value, a range, ''Unclear,''* or ''Not reported,'' as appropriate).	Overall sensitivity of 95%
D.4. Report precision data (a single value, a range, ''Unclear,''* or ''Not reported,'' as appropriate).	0.4 to 17%
D.5. Report specificity data (a single value, a range, ''Unclear,''* or ''Not reported,'' as appropriate).	Specificity varied from 14-70%,
D.6. Other performance measures reported.	The NNR value (number needed to read) ranged from 6 to 278
D.7. Other observations.	
**E. External validity testing (This section relates to testing the search filter on records that are different from the records used to identify the search terms.)**	
E.1. How many filters were tested for external validity on records different from those used to identify the search terms?	
E.2. Describe the validation set(s) of records, including the interface.	
For each filter report the following information.	
E.3. On which validation set(s) was the filter tested?	
E.4. Report sensitivity data for each validation set (a single value, a range, ''Unclear,'' or ''Not reported,'' as appropriate).	
E.5. Report precision data for each validation set (report a single value, a range, ''Unclear,'' or ''Not reported,'' as appropriate).	
E.6. Report specificity data for each validation set (a single value, a range, ''Unclear,'' or ''Not reported,'' as appropriate).	
E.6. Other performance measures reported.	
E.7. Other observations.	
**F. Limitations and comparisons**	
F.1. Did the authors discuss any limitations to their research?	
F.2. Are there other potential limitations to this research that you have noticed?	
F.3. Report any comparisons of the performance of the filter against other relevant published filters (sensitivity, precision, specificity, or other measures).	
F.4. Include the citations of any compared filters.	
F.5. Other observations and/or comments.	
**G. Other comments (This section can be used to provide any other comments. Selected prompts for issues to bear in mind are given below.)**	
G.1. Have you noticed any errors in the document that might impact on the usability of the filter?	
G.2 Are there any published errata or comments (e.g., in the MEDLINE record)?	
G.3. Is there public access to prepublication history and/or correspondence?	
G.4. Are further data available on a linked site or from the authors?	
G.5. Include references to related papers and/or other relevant material.	
G.6. Other comments.	
