# Supplementary material for: Development and evaluation of a search filter to identify prognostic factor studies in Ovid MEDLINE
Source: BMC Med Res Methodol. 2022 Apr 10;22:107. doi: 10.1186/s12874-022-01595-9 (PMC8996648; doi:10.1186/s12874-022-01595-9)
Supplement: Supplementary file 2 — Additional file 2. Terms analysed in word frequency analysis. [file 12874_2022_1595_MOESM2_ESM.pdf]

Additional file 2: Terms analysed in word frequency analysis

**Negatives (2203)**	**unique**	**title**	**abstract**	**keywords**	**points**		**Positives (31)**	**unique**	**title**	**abstract**	**keywords**	**points**
analysis	615	67	429	244	740		analysis	16	1	12	7	20
associated	665	83	642	0	725		associated	16	3	16	0	19
baseline	0	0	0	0	0		baseline	3	0	3	0	3
better survival	0	0	0	0	0		better survival	4	1	4	0	5
clinical	649	124	583	47	754		clinical	15	4	15	0	19
cohort	217	42	179	109	330		cohort	8	0	7	2	9
conclusion	417	0	417	0	417		conclusion	7	0	7	0	7
course	0	0	0	0	0		course	3	2	3	0	5
effect	296	60	259	4	323		effect	6	1	5	0	6
factor	236	43	211	47	301		factor	5	0	5	1	6
factors	748	129	513	410	1052		factors	11	2	7	6	15
findings	293	8	289	0	297		findings	7	1	7	0	8
followed	143	0	143	0	143		followed	7	1	6	0	7
hazard	0	0	0	0	0		hazard	4	0	4	0	4
hazard ratio	0	0	0	0	0		hazard ratio	4	0	4	0	4
healed	0	0	0	0	0		healed	4	0	4	0	4
healing rate	0	0	0	0	0		healing rate	3	0	3	0	3
identify	169	3	169	0	172		identify	5	0	5	0	5
measured	186	2	185	0	187		measured	5	0	5	0	5
mortality	0	0	0	0	0		mortality	6	1	5	3	9
multivariate analysis	0	0	0	0	0		multivariate analysis	5	0	4	2	6
outcome	499	44	301	312	657		outcome	9	2	3	6	11
outcomes	267	49	259	0	308		outcomes	4	1	4	0	5
predictive	0	0	0	0	0		predictive	3	0	1	2	3
predictors	0	0	0	0	0		predictors	2	2	2	0	4

prognosis	106	7	45	79	131		prognosis	9	0	3	8	11
prognostic	0	0	0	0	0		prognostic	6	3	5	0	8
prognostic factors	0	0	0	0	0		prognostic factors	2	1	2	0	3
prospective	238	56	171	181	408		prospective	5	0	4	3	7
prospective studies	191	0	14	181	195		prospective studies	4	0	1	3	4
recovery	0	0	0	0	0		recovery	1	1	1	1	3
retrospective	112	16	68	89	173		retrospective	8	0	2	7	9
retrospective studies	0	0	0	0	0		retrospective studies	7	0	0	7	7
risk	417	67	308	236	611		risk	5	1	4	3	8
risk factors	243	29	94	211	334		risk factors	2	1	1	2	4
role	268	54	208	42	304		role	7	2	5	0	7
severity	249	19	170	123	312		severity	3	0	1	3	4
survival	0	0	0	0	0		survival	9	3	9	3	15
time	0	0	0	0	0		time	10	0	7	5	12
time factors	0	0	0	0	0		time factors	5	0	0	5	5
treatment outcome	295	5	19	282	306		treatment outcome	6	0	0	6	6
worse survival	0	0	0	0	0		worse survival	4	0	4	0	4
