# Supplementary material for: Development and evaluation of a search filter to identify prognostic factor studies in Ovid MEDLINE
Source: BMC Med Res Methodol. 2022 Apr 10;22:107. doi: 10.1186/s12874-022-01595-9 (PMC8996648; doi:10.1186/s12874-022-01595-9)
Supplement: Supplementary file 3 — Additional file 3. Delphi Panel Results. [file 12874_2022_1595_MOESM3_ESM.pdf]

Additional file 3. Delphi Panel Results

	P 1	P 2	P 3	P 4	P 5	P 6	P 7	P 8	P 9	P 10	P 11	P 12	P 13	P 14	P 15	Mean
**predictors**	9	8	5	9	8	8	9	9	8	9	7	9	9	9	9	**9**
**prognosis**	9	9	7	9	8	9	8	7	9	8	7	9	9	9	9	**9**
**prognostic**	9	9	5	9	8	9	8	7	9	8	7	9	9	9	9	**9**
**prognostic determinants**	9	9	6	9	8	9	8	9	9	7	8	9	9	9	1	**9**
**prognostic factors**	9	9	5	9	8	9	9	9	9	9	9	9	9	9	1	**9**
**prognostic markers**	9	7	8	9	8	9	8	9	9	9	8	9	9	8	1	**9**
**prognostic variables**	9	9	7	9	8	9	8	9	9	9	8	9	9	9	1	**9**
**risk factors**	9	9	7	9	8	8	9	9	7	8	9	9	9	8	1	**9**
**causal factor**	8	8	3	7	9	8	8	9	7	6	7	7	8	9	1	**8**
**predictive**	6	7	5	8	8	8	8	9	9	8	8	9	8	9	9	**8**
**predictive markers**	9	7	5	8	7	8	8	9	8	8	8	9	9	7	1	**8**
**QUIPS**	9	7	7	9	8	9	8	8	8	7	8	5	9	8	3	**8**
**REMARK**	7	5	7	9	9	9	9	8	8	9	8	1	9	9	3	**8**
**TRIPOD**	8	6	9	8	9	9	9	8	8	5	7	1	9	9	3	**8**
**predictive factors**	6	9	7	9	8	8	8	9	8	9	9	9	9	8	1	**8**
**prognostic index**	8	8	9	9	8	9	8	9	9	9	8	8	9	8	1	**8**
**attributable risk**	7	7	3	8	7	7	9	8	8	5	7	6	8	8	1	**7**
**cohort**	9	4	3	7	8	7	7	9	8	6	9	1	7	8	8	**7**
**cox**	7	3	7	8	6	7	8	8	7	6	9	1	7	8	4	**7**
**exposure**	7	5	3	5	7	7	7	7	4	5	6	9	7	7	1	**7**

Additional file 3. Delphi Panel Results

**factor**	9	5	5	5	7	7	6	5	7	7	8	9	7	9	1	**7**
**factors influencing**	7	7	5	7	7	8	5	7	7	8	7	7	7	5	1	**7**
**factors of importance**	9	7	5	9	7	8	5	5	7	8	8	6	7	8	1	**7**
**hazard**	5	5	5	7	7	7	8	5	7	7	6	1	7	7	4	**7**
**hazard ratio**	5	5	5	7	8	7	8	8	7	8	7	1	7	7	2	**7**
**incidence**	3	4	5	8	8	8	7	4	9	5	9	1	7	8	8	**7**
**independent association**	9	6	5	7	7	7	7	9	5	8	1	8	5	5	1	**7**
**predictive model**	4	8	7	8	7	8	8	9	8	6	7	6	7	5	1	**7**
**risk**	9	7	9	8	7	7	7	8	5	7	7	6	7	7	5	**7**
**score**	4	7	7	5	7	7	6	8	4	3	8	1	7	8	5	**7**
**survival**	5	8	7	8	7	8	6	8	8	6	8	1	7	8	6	**7**
**worse survival**	7	6	9	8	7	8	6	7	8	5	9	1	6	8	2	**7**
**better survival**	5	5	3	8	7	8	6	8	7	5	7	1	6	6	1	**6**
**death**	4	5	6	7	7	8	5	7	9	3	9	1	5	6	5	**6**
**discrimination**	3	5	3	2	6	7	6	7	5	6	6	1	8	7	5	**6**
**influence**	9	5	5	2	7	6	5	6	5	7	9	5	6	8	1	**6**
**longitudinal**	5	5	5	7	7	7	7	6	6	6	5	1	6	7	4	**6**
**mortality**	4	6	5	7	7	8	4	6	9	4	6	1	6	6	5	**6**
**multivariable regression**	5	6	7	7	6	7	6	7	5	7	5	1	5	6	2	**6**
**role**	8	6	7	2	7	6	4	5	5	7	5	4	6	6	3	**6**
**treatment outcome**	1	7	9	2	7	8	3	8	1	4	7	1	6	7	1	**6**
**course**	3	4	3	2	7	8	5	1	9	3	7	4	7	7	8	**5**

Additional file 3. Delphi Panel Results

**severity**	6	4	8	7	7	7	3	4	4	3	4	1	6	6	5	**5**
**calibration**	2	5	3	2	6	6	6	5	4	6	5	1	6	6	1	**5**
**case-control**	7	4	3	2	6	5	5	7	2	5	3	1	5	5	6	**5**
**case-crossover**	4	5	3	2	5	5	5	5	2	5	6	1	5	5	1	**5**
**c-index**	5	5	3	2	6	7	7	8	5	6	3	1	8	7	1	**5**
**follow-up**	5	4	3	3	7	5	4	5	8	5	5	1	6	5	4	**5**
**harrell index**	5	3	7	2	5	5	6	7	2	5	3	1	6	5	1	**5**
**Hosmer-lemeshow**	2	4	5	2	5	5	6	8	5	5	5	1	6	5	1	**5**
**logistic**	5	4	5	7	6	6	6	1	5	6	6	1	5	7	4	**5**
**prospective**	5	5	7	2	7	6	5	8	2	6	3	1	6	6	5	**5**
**prospective studies**	5	5	7	2	7	7	5	8	2	6	5	1	6	5	1	**5**
**register**	2	5	7	2	7	5	6	1	2	5	5	1	6	6	1	**5**
**statistical models**	2	5	7	2	5	6	3	6	2	3	7	1	5	5	1	**5**
**time factors**	4	5	9	2	7	8	5	5	4	5	6	1	6	6	3	**5**
**worsen**	2	5	9	2	7	8	6	4	4	5	7	1	7	8	5	**5**
**associated**	6	6	3	2	7	7	3	7	1	6	3	6	4	5	1	**5**
**effect**	9	5	3	2	7	7	4	7	5	7	6	4	6	5	1	**5**
**multivariate analysis**	5	6	5	7	6	7	6	7	5	7	5	1	5	5	2	**5**
**AUC**	5	4	3	2	6	4	4	2	4	5	6	4	6	4	1	**4**
**baseline**	1	5	3	2	7	6	4	3	3	4	5	4	5	5	1	**4**
**case-population**	4	5	3	2	6	6	6	4	2	5	4	1	6	7	1	**4**
**cross- sectional**	3	4	3	2	4	5	4	5	1	4	3	1	4	4	6	**4**
**episode**	3	4	3	2	6	4	4	4	4	3	4	1	6	4	1	**4**

Additional file 3. Delphi Panel Results

**healed**	2	4	4	1	4	7	4	1	1	3	5	1	6	5	1	**4**
**healing rate**	2	4	3	1	4	7	5	5	1	3	5	1	6	5	1	**4**
**identify**	6	5	3	2	6	8	3	1	4	3	5	1	5	6	1	**4**
**impact**	8	4	3	3	7	8	3	1	5	7	3	4	6	7	1	**4**
**model and validation**	1	4	7	2	5	4	4	5	2	4	8	5	6	5	2	**4**
**recovery**	2	4	7	2	4	5	4	6	3	3	5	1	6	4	1	**4**
**retrospective**	2	4	5	2	5	5	4	6	1	6	4	1	4	4	3	**4**
**retrospective studies**	2	4	7	2	4	4	4	6	1	6	6	1	4	4	1	**4**
**outcome**	1	7	5	2	7	6	3	8	1	4	7	4	5	4	2	**4**
**analysis**	1	4	3	2	5	6	3	5	1	1	3	1	4	5	1	**3**
**assessment outcomes**	1	5	3	2	5	8	3	7	1	3	5	1	6	4	1	**3**
**clinical**	2	3	3	1	5	3	3	1	1	3	1	1	4	3	1	**3**
**conclusion**	2	3	3	1	3	3	3	1	1	1	3	1	4	4	1	**3**
**findings**	3	3	3	1	4	4	3	2	1	1	3	4	4	4	1	**3**
**followed**	2	3	5	2	4	5	3	1	8	5	3	1	6	4	1	**3**
**harms**	5	4	3	3	6	7	5	3	1	3	2	1	5	6	1	**3**
**measured**	2	3	3	1	3	6	3	1	1	4	3	1	4	4	1	**3**
**time**	1	3	7	1	6	3	3	4	1	5	3	1	6	4	1	**3**

*P1-P15: Each column is a different Delphi panellists ratings.
